# Integrative Network Biology: Graph Prototyping for Co-Expression Cancer Networks

**DOI:** 10.1371/journal.pone.0022843

**Published:** 2011-07-29

**Authors:** Karl G. Kugler, Laurin A. J. Mueller, Armin Graber, Matthias Dehmer

**Affiliations:** Institute for Bioinformatics and Translational Research, UMIT, Hall in Tyrol, Austria; University of New Orleans, United States of America

## Abstract

Network-based analysis has been proven useful in biologically-oriented areas, e.g., to explore the dynamics and complexity of biological networks. Investigating a set of networks allows deriving general knowledge about the underlying topological and functional properties. The integrative analysis of networks typically combines networks from different studies that investigate the same or similar research questions. In order to perform an integrative analysis it is often necessary to compare the properties of matching edges across the data set. This identification of common edges is often burdensome and computational intensive. Here, we present an approach that is different from inferring a new network based on common features. Instead, we select one network as a graph prototype, which then represents a set of comparable network objects, as it has the least average distance to all other networks in the same set. We demonstrate the usefulness of the graph prototyping approach on a set of prostate cancer networks and a set of corresponding benign networks. We further show that the distances within the cancer group and the benign group are statistically different depending on the utilized distance measure.

## Introduction

For many diseases no longer single genes act as marker, but a set of interacting genes may be used to characterize or diagnose a pathological process [1]. Driven by that rational a plethora of new data analysis methods emerged over the last years, as the need for methods that are able to capture the related complexities arose. A simple example is to look for objects that are highly connected to other objects and may therefore play a central role in regulatory processes. The network-based analysis [2] of biological data is one related field in systems biology [3]. Whereas classical data analysis was driven by a reductionistic point of view, modern network biology aims at perceiving the data holistically [3]. Using networks allows leaving behind the static exploration of one feature at a time, and enabling an investigation of the more realistic dynamic nature of biological and medical data. The dynamics lie in several dimensions, as systems change over time [4], react to perturbations [5] or are simply made up by biological functions, which are interlinked into complex cascades [6]. Simultaneously, combining different data sources has become a standard procedure in modern computational biology. Be it by means of data integration or classical meta-analysis, much effort is still being put into standardizing approaches that enable an integrative analysis [7]. Integrative approaches allow increasing the evidence base for new findings by combining information from different sources. In a classical view data integration refers to the integration of data of different nature (e.g. gene expression and proteomics). In this present paper, we also refer to the integration of the same type of data as data integration.

Research for combining network biology and integrative data analysis has flourished over the last years [8]–[10]. This allows deriving generalizations from a set of differing networks that investigate the same or similar research questions. Such general findings can be used for answering biological questions or for creating new hypothesis about underlying processes. Measuring the similarity between networks has been proven useful for assessing systematic effects of time course for metabolic networks [8], matching regulatory interactions [9] or for identification of similar subgraphs in pairs of networks [10]. Another application of comparative network analysis is the systematic comparison of two association networks that were trimmed for partial correlations [11]. Yet, detecting and inferring knowledge about common properties for a set of networks is a challenging task since comparing networks depends on the definition of the underlying similarity measure. However, the similarity between any objects is not uniquely defined since multifaceted aspects such as structure, function and semantics are involved [12]. Therefore, it is necessary to find comparable features in biological networks. Often this is done by detecting common edges or vertices, and comparing them or their distributions [13], [14]. To address the issue of meaningfully comparing biological networks a multitude of methods has been developed. We can here only present a small selection of these approaches and their applications. Piruzian et al. employed topological information for integrating transcriptomic and proteomic data in a rank-based approach [15]. A generalized form of the degree distribution, the so called graphlet degree distribution, can be applied for determining network similarity [16]. Graphlets were also used to align PPI networks from human and yeast [17]. A statistical method for comparing large disease networks inferred from cervical cancer using a tree decomposition and alignment technique was also proposed in [18]. Here, we focus on the application of comparing networks, that are derived from the same type of data and are used as representations for a class of specimen. Therefore, we analyze a set of association networks derived from prostate cancer gene expression data. By making use of this combination it is possible to derive generalized information about the network-based findings related to certain diseases or developmental states. A common approach to the problem of analyzing network properties by means of meta-analysis is to compare the overlap of edges in different networks. We demonstrated its usefulness for a network-based integration in a previous study [19]. A similar approach for shared edges was given by Cootes et al. [10]. An alternative method was presented by Wang et al., who utilized information about the effect-size to combine information from a set of network [20]. However, this approach requires information about the effect-size to be available. Detecting common edges in a network is a challenging task if no proper mapping between the vertex labels is available. When considering co-expression networks, the vertex labels refer to gene names. In order to generate a common name space across the different networks, it is therefore useful to map the study-specific, platform-depended gene identifiers to other identifiers, e.g. Entrez gene identifiers.

**Figure 1:**
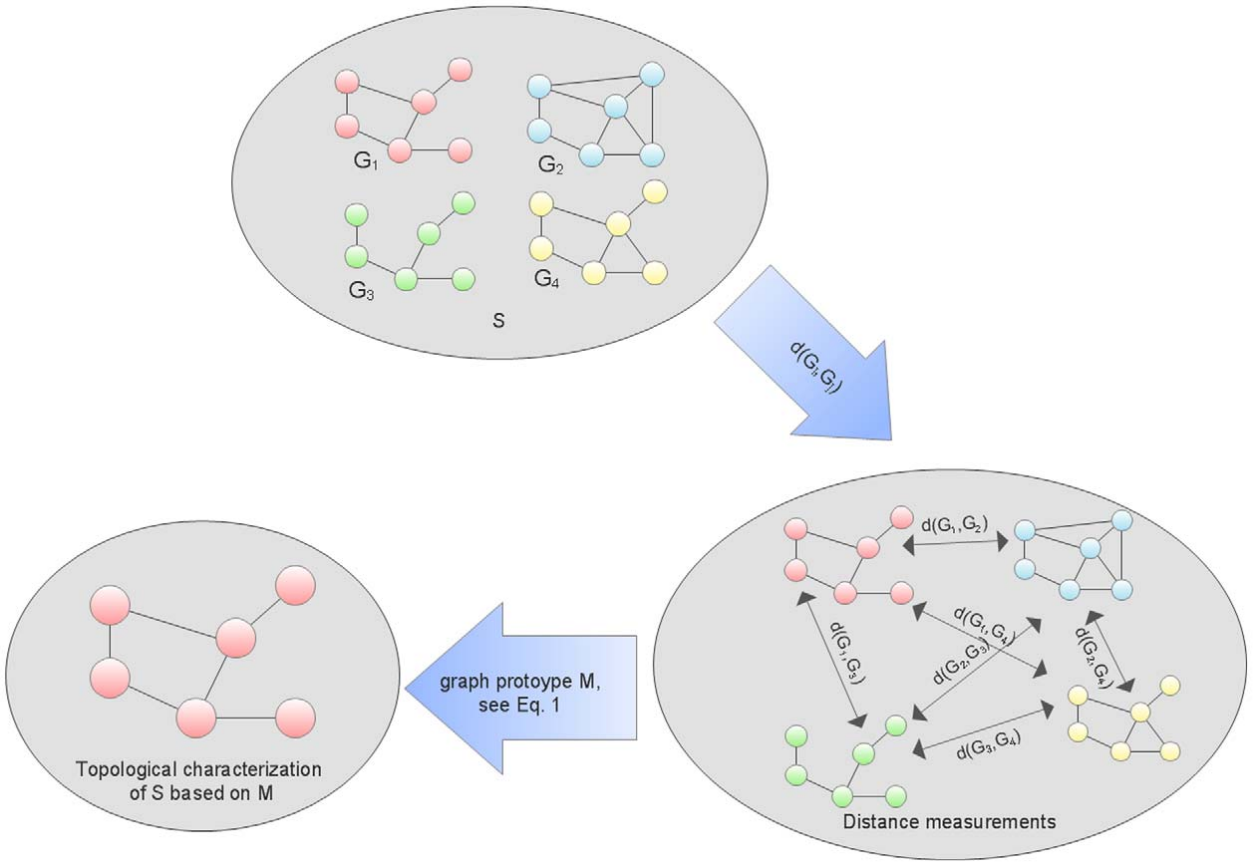
The graph prototyping method. This figure schematically illustrates the derivation of the graph prototype.

In the present paper we demonstrate an alternative approach for inferring common topological properties for a set of networks. Here, graph prototyping can be understood as a method that selects an existing network from a set of networks as a representative for the complete set, with respect to an underlying graph distance measure [21]. This means that the structural graph prototype represents the topological properties of a complete set of networks, depending on the selection criterion that is defined by the graph distance measures. A schematic illustration for selecting a graph prototype is given in Fig. 1. Note that other definitions of graph prototypes such as the so-called consensus tree [22] have been also explored. But those won't be discussed in this paper. Thus, this prototype network can then be used for performing a topological analysis and inferring new knowledge, as it represents the properties of all other networks from the same set. One strong-point of this method is that detecting common edges or nodes may become unnecessary, depending on the employed graph distance measures. Then, it is crucial using a graph distance measure whose computational complexity is polynomial. To implement graph prototyping, we select proper graph distance measures that are able of meaningfully quantifying the distance between two networks. As part of our contribution we describe four distance measures that are based on the probability distributions of network properties. This is another strong-point of this method, as it can be modified to make use of other, customized graph distance measures. To demonstrate the selection of a graph prototype [21], [23] we make use of prostate cancer gene expression studies. 25% of newly diagnosed male cancers in the US are prostate cancers [24], which makes it an attractive target for ongoing biomedical research. A broad range of studies have been conducted over the last years, and much of the corresponding data is available in public data repositories [25]–[27]. We apply our method on a set of seven prostate cancer studies [28–24], which consist of cancer samples and samples from benign or healthy tissue. We expect a two-fold result: First, we expect to see significant structural differences between benign and cancer studies by making use of topological measures. Secondly, we expect to see significant differences between the distances within the cancer data networks and the distances within the benign data networks. This could show that not only the networks themselves differ, but that even the similarities between the two groups differ. If so, the pathogenic processes that are caused by the cancer are most likely responsible to explain these observations. Based on previous work [19] we expect to observe higher similarities within the cancer group. More precisely, we expect distances within data sets from a cancer group to be smaller than those from a benign set.

The paper is organized as follows: In the ‘Data and Methods’ section we present the exploited data sets and the inference process of the networks. Then, we describe the graph prototyping approach and the employed graph distance measures in detail. The section ‘Results’ summarizes and describes the obtained results. The section ‘Discussion and Outlook’ ends the paper with discussing our results and is followed by some final remarks.

## Materials and Methods

### Prostate Cancer Data

We demonstrate the graph prototyping approach using a set of prostate cancer studies. Since this cancer has been thoroughly investigated for the last years, a larger number of gene expression data is on-hand through public repositories. For the presented study a survey on the repositories NCBI GEO [25], EBI Arrayexpress [26] and Oncomine [27] was conducted. For inclusion into our analysis studies have to report gene expression levels from prostate cancer and benign specimen using microarrays. Benign specimen are either samples from normal tissue adjacent to tumors or healthy males. We expurgate metastatic forms from the cancer samples for this study in order to decrease heterogeneity in the networks. Cell line expression data was also excluded. To reduce the data preparation and mapping effort we only include Affymetrix microarray platforms in this study.

For conducting this analysis we select seven data sets [28]–[34] from the data pool as listed in Table 1. To investigate the effect of sample size within the studies on our results a broad range of sample sizes (from small studies to larger ones) is allowed. After the selection of studies to be included, we re-perform microarray preprocessing. The given sample sizes in Table 1 refer to the post-quality control state. To enable inter-study comparison of the genes, the original identifiers are mapped to Entrez gene identifiers by using the biomaRt package [35] for Bioconductor [36]. Wherever multiple probesets map to one Entrez gene identifier, we retain the measurement with the highest variance. After this mapping 8906 genes common within all seven studies are left for further analysis. For deriving a suitable network representation of the data, the creation of association networks was chosen. However, the methods presented below are applicable to a range of other network types too, if adopted properly.

**Table 1:** The data sets that were used in this study.

Name	Journal	Year	Platform	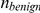	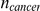
Chandran	BMC Cancer	2007	Affymetrix GeneChip HG U95Av2	15	57
Liu	Cancer Res	2006	Affymetrix GeneChip HG HG-U133A	13	41
Wallace	Cancer Res	2008	Affymetrix GeneChip HG U133A 2.0	14	68
Tsavachidou	J Natl Cancer Inst	2009	Affymetrix GeneChip HG HG-U133A	49	23
Singh	Cancer Cell	2002	Affymetrix GeneChip HG U95Av2	48	50
Yu	J Clin Oncol.	2004	Affymetrix GeneChip HG U95Av2	58	59
Wang	Cancer Res	2010	Affymetrix GeneChip HG HG-U133A	3 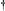	138

We infer the networks from public available data sets. The given sample size are after quality control and related filtering. 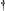 We do not infer a network from this group, due to the small sample size.

### Network Inference

To infer a proper network representation of the underlying data is an important challenge in network-based research [37]–[39]. A broad range of network representations for biological data exist [39]–[41], and the graph prototyping method presented hereinafter can be applied for most of them. Here, we utilize information about the association between two genes. The resulting networks are therefore called association networks. For inferring and analyzing gene expression data as association networks, co-expression relationships have been often utilized [42]. Note, that association does not necessarily indicate causality. One way to address this problem is to apply the concept of causal memberships [43], where genes have been functionally categorized.

Here, we utilize the mutual information as a measure for the association, as described in [39]. For inferring the networks from the gene expression data, we make use of the MRNETB algorithm [38]. To set up data sets for selecting a graph prototype, we infer two networks from each study. One network that is based on the information from the benign samples in a study, and one network from the cancer samples in the same study. This leads to 6 benign networks, and 7 cancer networks, as we remove the benign network from the Wang data. This is done due to the small sample size (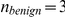) since we regard the inferred network as being of little reliability. In general, inferring a network for each patient group separately allows performing topological comparisons and thereby deriving new insights on the underlying functional differences.

### Selection of a Graph Prototype

To generalize the graph similarity problem [21], it has been shown by Dehmer et al. that one graph can be used to represent a set of other comparable graphs [21]. The task of determining this so called graph prototype can be solved by applying distance or similarity measures [21], [44]. Let 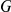 be a network, and 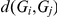 be a graph distance measure. Having a set of networks 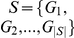, the graph prototype can be expressed by [21], [23], [45]:
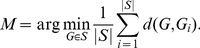
(1)We see that 
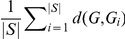
 in Eq. 1 gives the mean distance from network 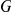 to all other networks in 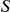. We denote this as 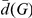. Our goal in the present paper is to apply a selection of graph distance measures for selecting graph prototypes from a set of prostate cancer networks and a set of corresponding benign networks. Applying different graph distance measures means that we can cover different aspects of structural similarity. In general, it is a still outstanding problem what aspect of structural similarity an underlying measure captures [44]. If different graph distance measures select the same network as a graph prototype for a set of networks, this increases the validity of the selection. With respect to the employed distance measure the graph prototype represents the topological properties of the other networks from the same set 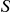. It can therefore be used for performing a topological and functional analysis.

### Graph Distance Measures

In order to perform graph prototyping it is necessary to meaningfully measure the distance between two networks. In this subsection we present two approaches for accomplishing this task. The first approach is based on using inexact graph matching. In particular, we choose the so-called graph edit distance (*GED*) [46]. The second approach is based on comparing two discrete probability distributions [47], that are inferred by deriving structural features of the networks.

The *GED* is the minimum cost of a sequence for transforming a graph 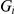 into another graph 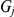 using edit operations (deleting and inserting edges or deleting, inserting, and substituting vertices) [46]. The underlying problem (to compare two graphs structurally) can be seen as a generalization of Levenshtein's method [48] for comparing strings. Generally, calculating the *GED* for (unlabeled) graphs is computationally demanding, as it is NP complete [49]. For our purpose the complexity can be reduced due to three facts [50]: i) All of our networks 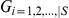 have the same number of (unconnected) vertices 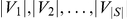, ii) all the vertices are labeled uniquely, and iii) by selecting only the genes that are present in all studies, all the networks have the same set of vertices, which frees us of deleting, inserting or substituting any vertices. Thus, reducing the computational complexity to 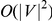 [49]. For measuring the distances between two networks, we employ a normalized form, which is given by the percentage *GED* (*pGED*) [51]:
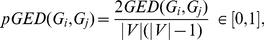
(2)where 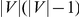 is the number of maximum possible edges in 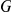, and the factor 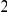 refers to the non-directed nature of the edges. We weight all remaining edit transformations (insert, delete) equally by assigning a weight of 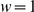.

An information-theoretic approach for quantifying distances between graphs can be defined based on the Kullback-Leibler divergence (*KLD*) [47]. We define two discrete probability distribution 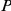 and 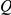, so that the *KLD* is given as [47]:
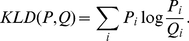
(3)The *KLD* is always defined positively for the distance between 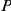 and 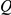. Note, that 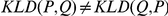. As the *KLD* is asymmetric and does not satisfy the triangle inequality, it is no metric [52]. We then calculate the graph prototype by setting 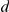 to the *KLD* in Eq. 1. Numeric stability is ensured by setting probabilities of zero to 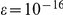.

**Figure 2:**
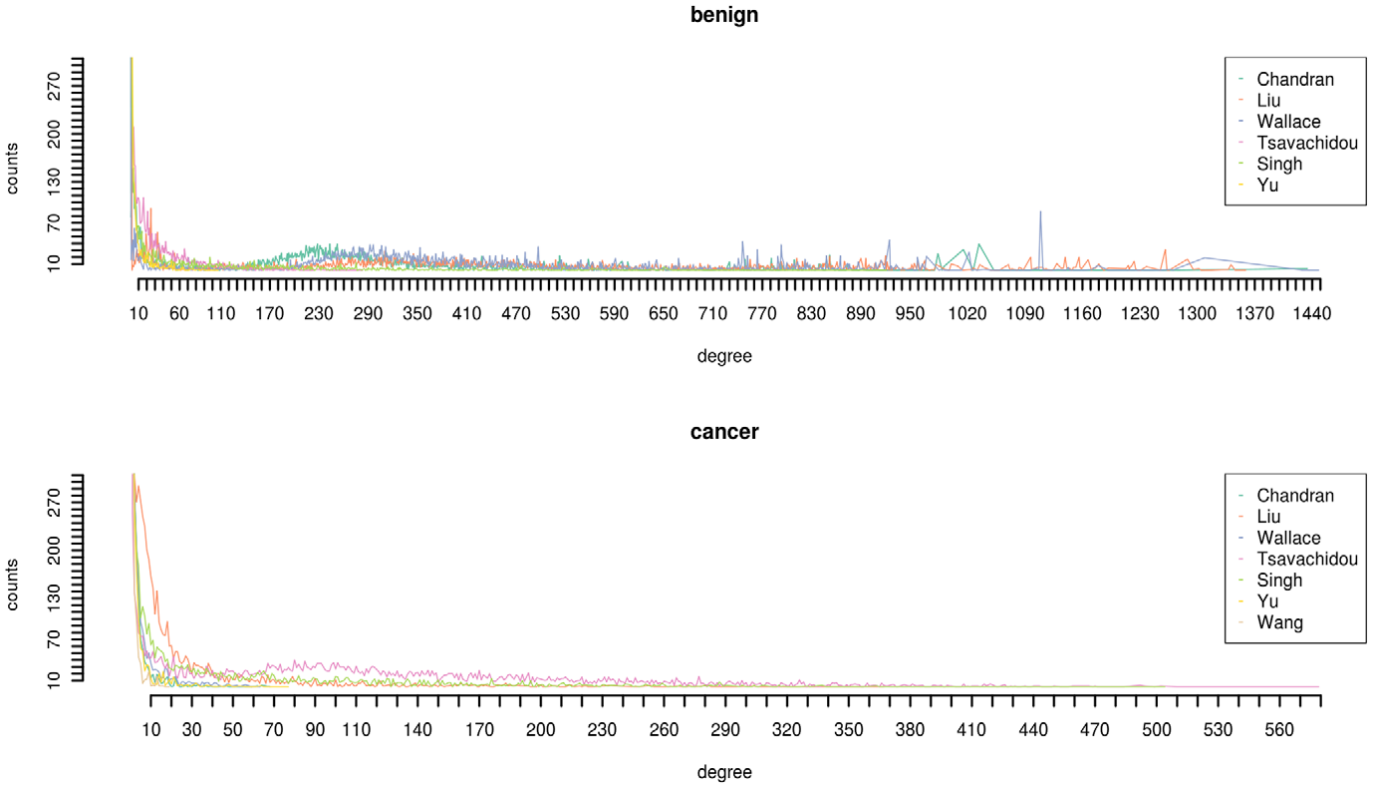
The degree distributions. The degree distributions for the benign data (top) and the cancer data (bottom). For displaying reasons we trimmed the number of counts at 300.

A typically distribution that is often used in Systems Biology is the degree distribution 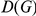. In undirected networks, the degree 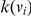 gives the number of neighbors for a vertex 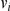. If we define 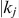 to be the number of vertices with 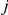 neighbors, we can derive a probability distribution so that:
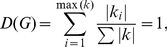
(4)where 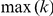 is the maximum number of neighboring vertices in 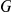. Fig. 2 shows the degree distributions of the benign and cancer networks. 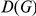 can be used to characterize a network [9], [42], [53]–[55], and has been shown to be scale-free and follow a power-law distribution for various types of biological networks [42], [53]–[55]. Power-law distributions of the degrees can also be seen in Fig. 2. Here, we use 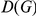 to calculate the *KLD*, which we therefore denote as 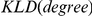.

**Figure 3:**
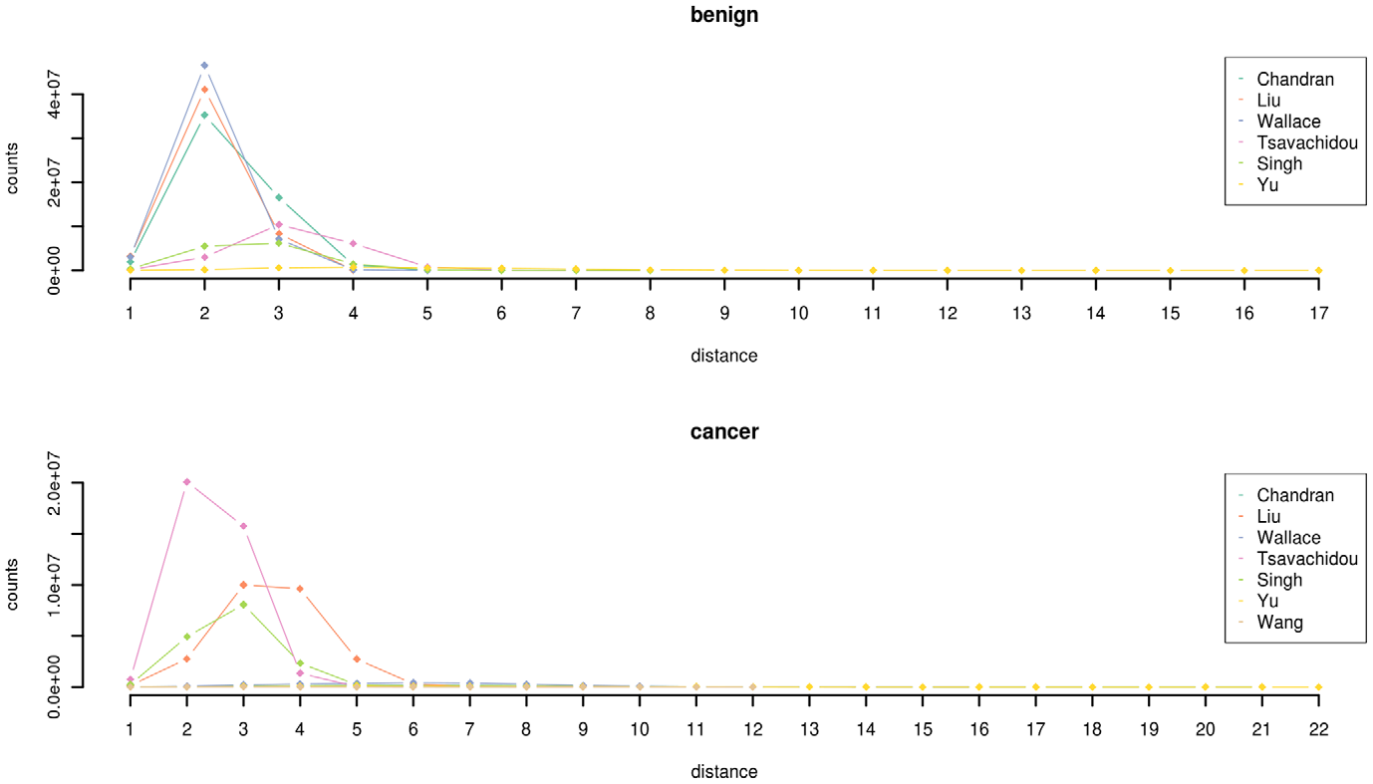
The distribution of distances. The distance distributions for the benign data (top) and the cancer data (bottom).

Distances present another prominent network invariant. For a vertex 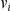 the distance to all other vertices is given by
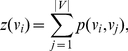
(5)where 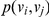 is the shortest path between the vertices 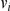 and 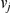. If we let 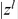 be the cardinality of all the distances with the length 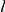, then the according distance distribution 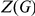 is given as
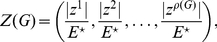
(6)where 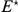 is the number of paths. We see that 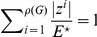. Note, that 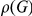 is the diameter of 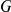, which is the maximum of the shortest paths between all pairs of vertices. The distance distributions for the networks is presented in Fig. 3. We employ the distance distributions of the included networks in order to quantify the distance between two networks, which is denoted as 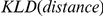.

While for the three distance measures that we presented above the complete, unconnected network was analyzed, we now present two distance measures that work on connected graphs only. This means that we have to infer the largest connected subgraph of each network and apply the two distance measures to them. The third distribution that we include in our *KLD*-based distance measures is based on vertex probabilities [56]. A vertex probability 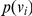 assigns a probability value to a vertex 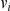 by making use of a so called vertex functional 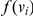 [56]:
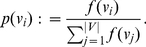
(7)We see that 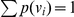. In this paper we utilize the following vertex functional [56]:
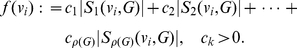
(8)The number of vertices in the 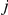-th sphere is given for every vertex 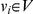 as 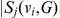 [56]. We see that 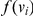 is based on metrical properties of graphs [57]. Here, we let the weighting factors 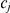 decrease in an exponential manner. This allows us to emphasize the vertices fairly close to 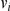, as they are probably stronger effected by information that spreads out from 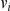 [56].

Finally, we use a distribution that can be calculated by using the topological information content based on vertex orbits [58], [59]. An orbit contains topologically equivalent vertices [58], and 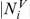 provides information about the number of vertices belonging to the 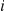-th vertex orbit [58]. We here determine a probability distribution by summing up the number of orbits sharing the same number of vertices within a network 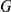. Let 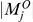 be the number of orbits containing 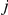 vertices. If 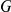 has 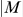 vertex orbits then we obtain the orbit distribution
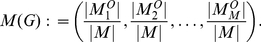
(9)Note, that 
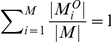
, where 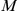 is the sum of the number of orbits containing the same number of vertices. The information about the distribution of topological equivalent vertices in each of our networks can then be used to combine the information for a set of networks by the 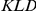. We refer to this as 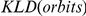.

With each of these four presented probability distributions we can cover different aspects of topological properties of our networks. The probability distribution for 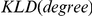 is based on information about how connected the genes in each of the networks are. Information about the communication distances between genes is reflected by the distribution that is used in 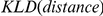. 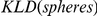 is based on a probability distribution that describes the spread of information in a network, while the probability distribution in 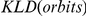 reflects topological equivalence of vertices. Table 2 summarizes the employed distance measures. After having introduced our formal apparatus, we compute the distances and graph prototype for the two sample groups (benign and cancer). For calculations and statistical analysis we make use of the statistical programming language R (http://www.r-project.org). The probability distributions to calculate 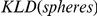 and 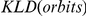 are computed using the QuACN package [60].

**Table 2:** The employed distance measures.

Name	Type	Description
*pGED*	Normalized graph edit distance	Minimization of a sequence of morphological graph edit operations that are needed to make two networks isomorph [46].
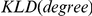	Kullback-Leibler divergence	Comparison of the degree distributions of two networks.
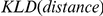	Kullback-Leibler divergence	Comparison of the distance distributions of two networks.
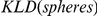	Kullback-Leibler divergence	Comparison of the sphere-based vertex probabilities of two networks.
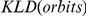	Kullback-Leibler divergence	Comparison of the distribution of the number of topologically equivalent vertices of two networks.

Here, we list the 5 distance measures that were used for the selection of a graph prototype.

## Results

**Figure 4:**
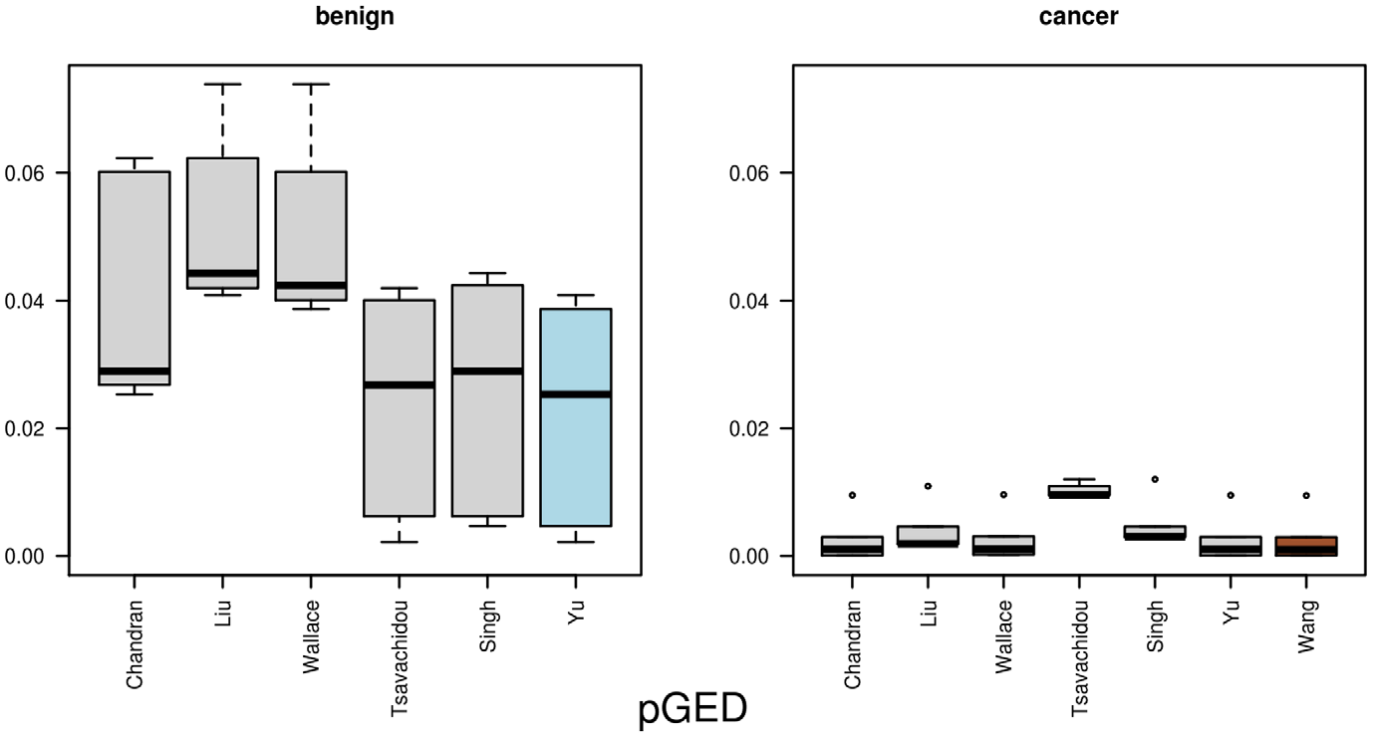
The results for *pGED*. This figure illustrates the distances from one network to all other networks, based on the normalized Graph Edit Distance *pGED*. In the left part it depicts the distances between one benign network and all other benign networks, whereas in the right part it lists the distances for one cancer network to all other cancer networks. The networks that are selected as graph prototypes are highlighted in different colors (benign = blue, cancer = brown).

**Figure 5:**
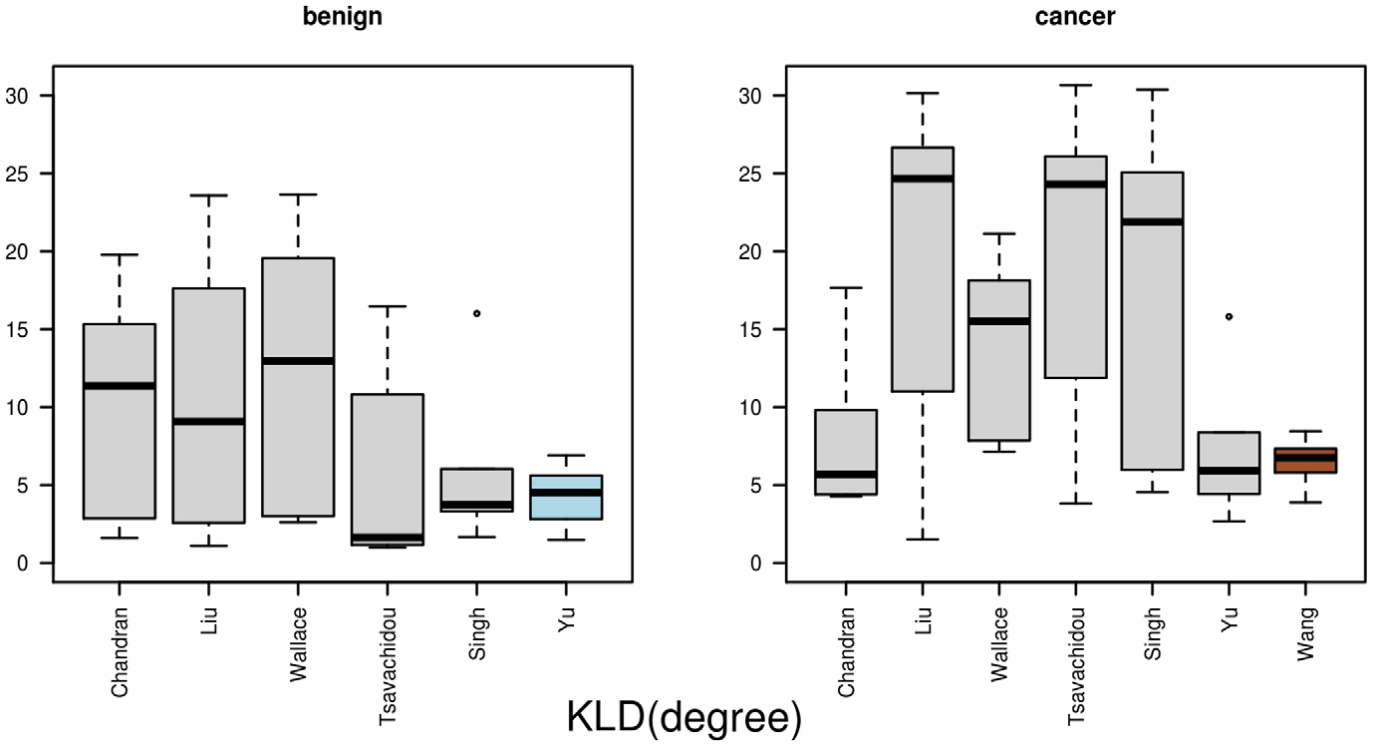
The results for *KLD(degree)*. Here, we show the distances between one network and all other networks as boxplots, measured by the Kullback-Leibler divergence, which was based on the degree distribution. In the left part we show the benign data, and in the right part the distances from the cancer data. The graph prototypes are highlighted.

**Figure 6:**
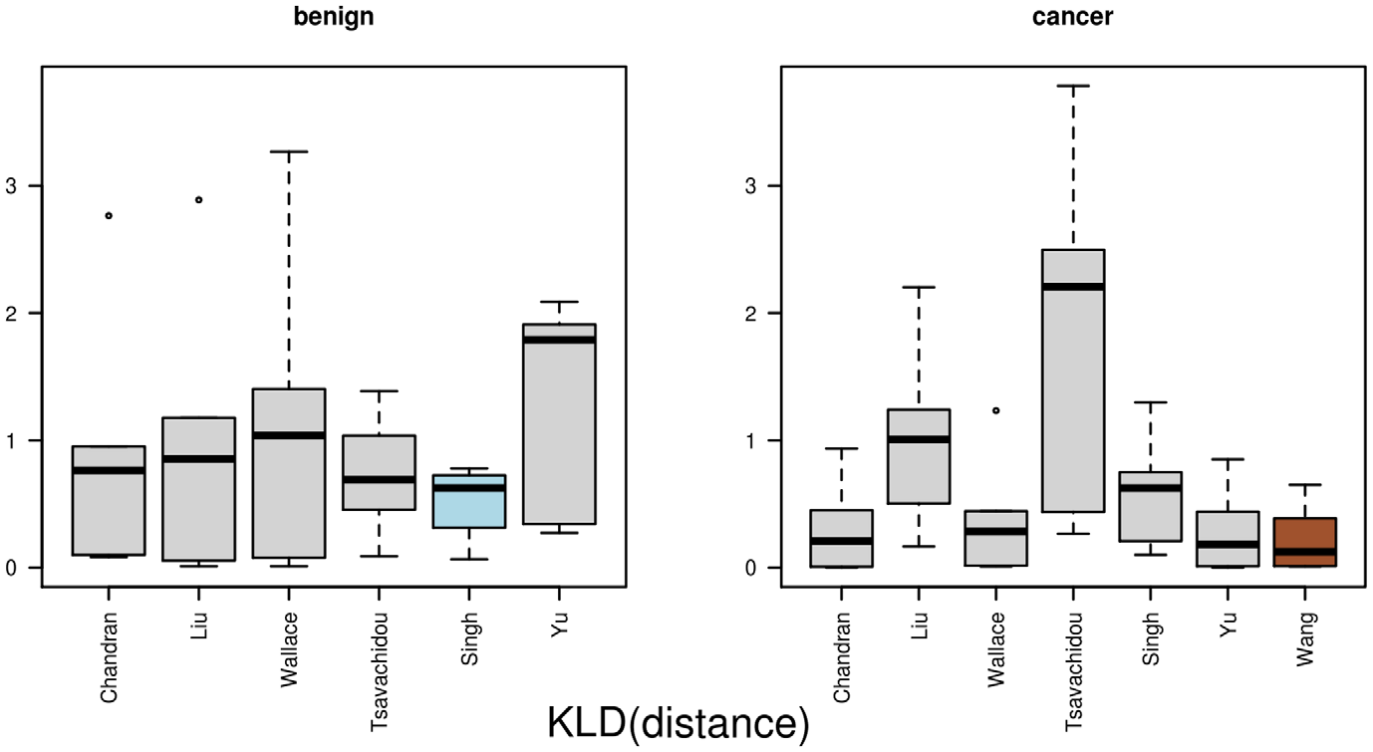
The results for *KLD(distance)*. This figure displays the distances between the networks as boxplots. The distances are based on the distribution of distances between vertices and the Kullback-Leibler divergence. In the left part are the distances between the benign networks, and in the right part the distances between the cancer networks.

**Figure 7:**
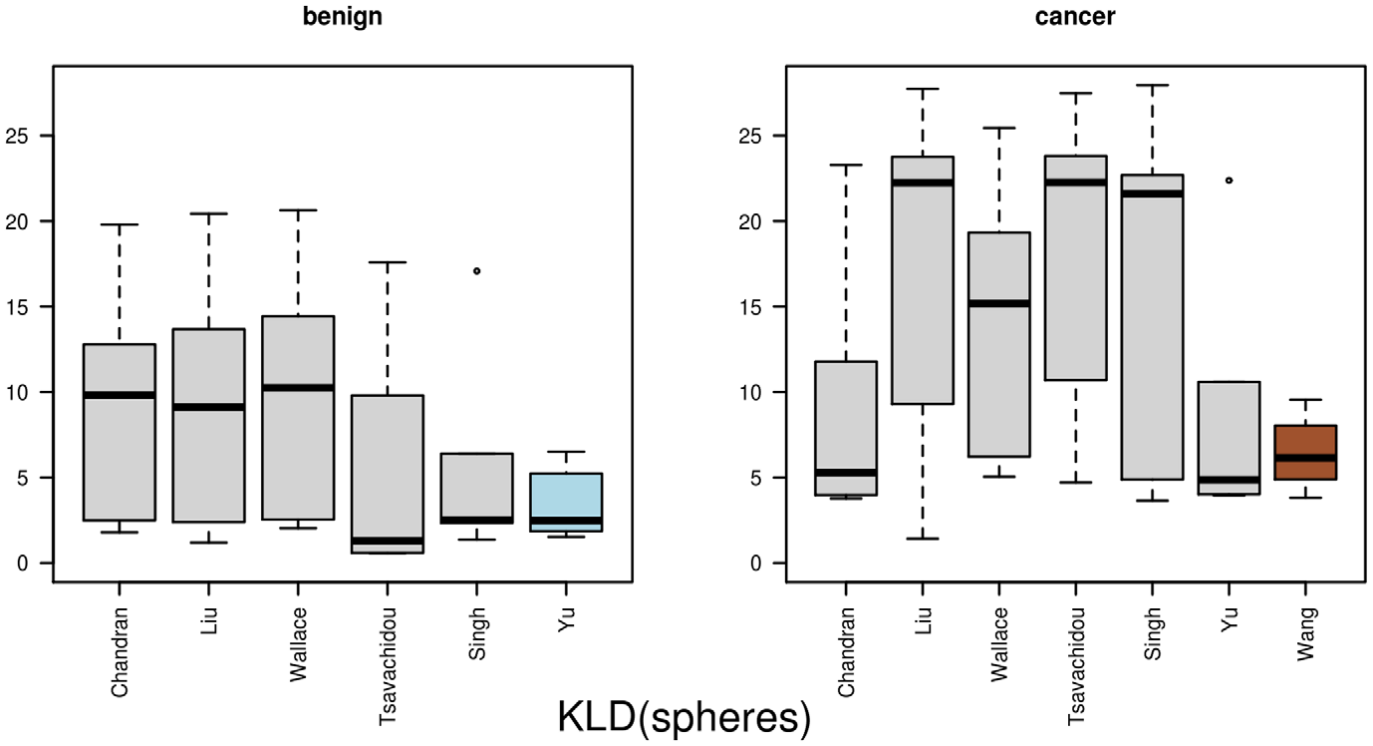
The results for *KLD(spheres)*. Here, we display the distances based on the Kullback-Leibler divergence, based on the sphere vertex functionals. In the left part we show the benign samples and in the right part the distances for the cancer samples. The selected graph prototypes are highlighted.

**Figure 8:**
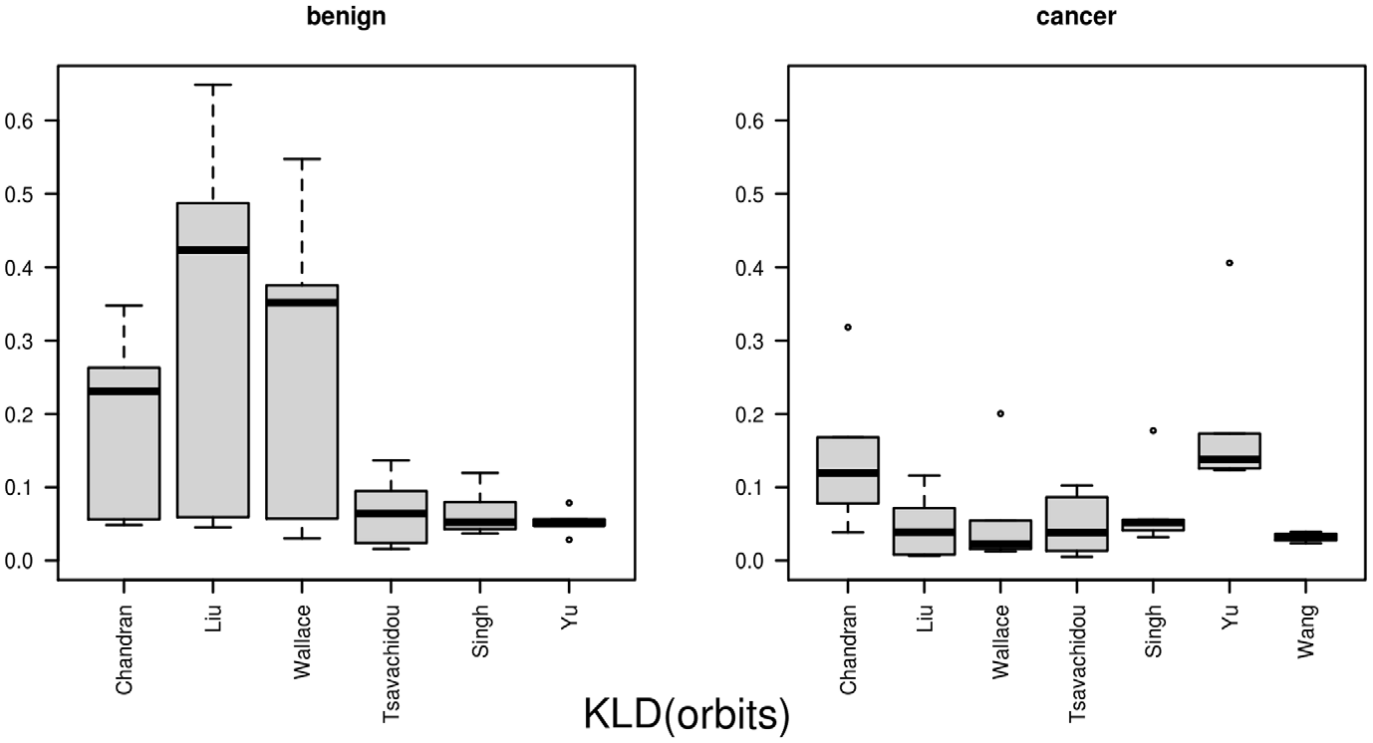
The results for *KLD(orbits)*. This figure illustrates the Kullback-Leibler divergences for the orbit probability distributions. In the left part it lists the benign samples, and in the right part the cancer samples from the studies.

Table 3 provides a summary of the mean distances for the five distance measures and the two groups. When calculating the 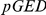 we see that the mean distance 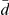 for the six networks ranges from 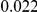 to 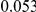 in the benign group, and from 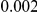 to 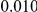 for the seven networks in the cancer group. The mean values are 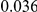 (benign) and 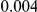 (cancer). Fig. 4 provides an illustration of all the single distances from one network to all others in the same group. A distinction between the distribution of 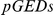 between the cancer and benign sample can be seen. For the benign group, the network that is based on the data by Yu is selected as graph prototype, while for the cancer group the network form the Wang data is selected. The mean distance for the Yu data is 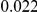 and for the Wang data 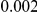. The network-specific mean distance 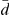 of the 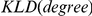 ranges from 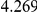 to 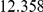 for the networks from the benign data, respectively 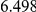 to 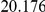 for the prostate cancer data. The mean values are 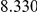 (benign) and 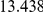 (cancer). Fig. 5 visualizes the results. The selected graph prototypes are Yu (benign) with a mean distance of 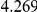 and Wang (cancer) with a mean distance of 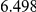. 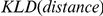, which is based on the distance distribution within a network, selects the networks from the Singh data (benign) and Wang data (cancer) as graph prototypes. The graph prototypes have a mean distance 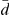 of 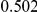 (benign) and 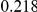 (cancer). The mean distances from one network to all others in the same groups for each set are 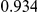 (benign) and 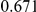 (cancer). The detailed results are depicted in Fig. 6. The networks from Yu (benign) and Wang (cancer) are again selected as graph prototypes when using 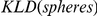. The minimum 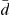 is 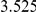 for the benign graph prototype, respectively 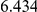 for the cancer graph prototype. The mean values are 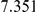 (benign) and 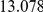 (cancer). The distances from one network to all other networks within the same group are illustrated as boxplots in Fig. 7. Together with the 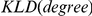 this represents the two cases, where the distance within the cancer data is larger then within the benign data. For the measure based on the orbits 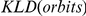 the distances of the graph prototypes are 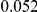 for the benign Yu network and 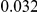 for the cancer network that is based on the Wang data. The mean distances are 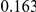 (benign) and 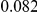 (cancer), as shown in Fig. 8.

**Table 3:** Ranges of mean distances 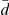.

	benign			cancer		
	min	mean	max	min	mean	max
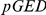	0.022	0.036	0.053	0.002	0.004	0.010
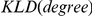	4.269	8.330	12.358	6.498	13.438	20.176
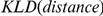	0.502	0.934	1.280	0.218	0.671	1.900
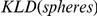	3.525	7.351	9.979	6.434	13.078	18.534
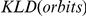	0.052	0.163	0.333	0.032	0.082	0.184

For each distance measure that is applied, we here list a summary of the results, based on the mean distances 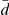 from one network to all other networks belonging to the same group (benign or cancer). This table shows the corresponding range and the mean values.

Our main hypothesis is that there is a significant difference between the distances in the group of cancer samples and the distances in the group of benign samples. For testing this hypothesis we employ a Wilcoxon test (see Table 4) for each of the five distance measures on the set of distances from the cancer samples and the benign samples. We correct for multiple testing with the Bonferroni method. 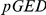, 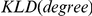, 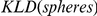 exhibit a significant difference (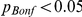), as can be seen in Table 4. The observed results support the hypothesis, see boxplots in the related figures.

**Table 4:** Wilcoxon tests on distance measures results.

	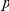	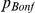	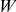
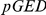	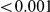	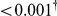	92
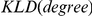	0.004	0.018 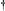	883
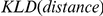	0.114	0.570	491
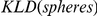	0.001	0.005 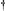	914
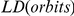	0.032	0.158	442

In order to test whether we could really see statically significant differences between the distances in the cancer network distances and the benign network distances we apply two-sided Wilcoxon tests. 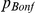 reports the p-values after multiple hypothesis correction as suggested by Bonferroni. 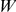 is the test statistic. 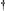 indicates a significant difference between the distances within cancer networks and the distances within the benign networks (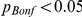).

**Figure 9:**
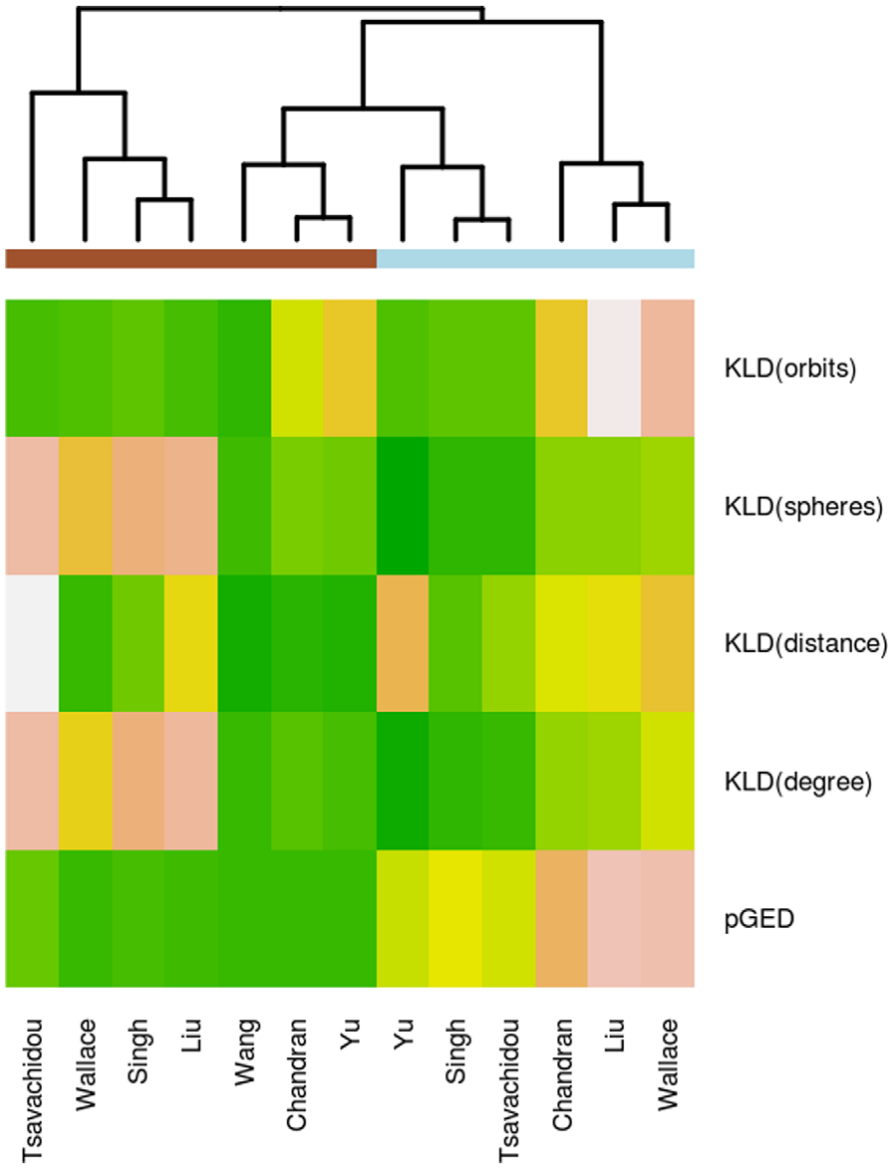
Heatmap of mean distances. We here show the mean distance from one network to the other networks within the same group (benign or cancer). For clustering we then omitted the group information. We independently add the group information as brown bars (cancer) and blue bars (benign).

For detecting patterns within the set of distances we employ clustering. Therefore, we normalize the result of each distance measure without the group information. This is done for each distance measure separately, so that the minimum of each distance measure is set to 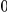 and the maximum to 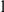. Then we apply hierarchical clustering. For each network we have a feature vector, that consists of the mean distance to all other networks for each of the five utilized distance measures. So, for the overall clustering we have a matrix with 5 rows and 13 columns. The corresponding heatmap, using the Euclidian distance and complete linkage, is depicted in Fig. 9. We also applied average linkage as clustering function, which lead to the same result. We therefore regard the observed outcome as stable with respect to these two linkage functions. The results show that three of the cancer networks (Tsavachidou, Wallace, Singh, Liu) form a separate cluster, while all other networks are clustered together. In the second cluster we observe that three of the cancer networks (Chandran, Wang, and Yu) cluster closely to three benign networks (Yu, Singh, Tsavachidou).

Based on the results from the graph prototyping we select the network from the Yu data as graph prototype for the benign set, and the network from the Wang data as graph prototype for the cancer set. For the analysis of the topological properties of the networks we investigate the hub genes. The distribution of the 15 mostly connected hub degrees is shown in Table 5. We observe that the main hub genes in the cancer network are remarkably smaller than those in the benign network. This is in accordance with known results for which we applied edge vote counting for the integrative network analysis [19]. In that study we also observed fairly small degrees in the common cancer network. A dysregulation of hub genes, associated with the cell-cycle, may play an major role in the development of an aggressive form of prostate cancer [61]. Similar to other scale-free networks [62], [63], biological networks may be vulnerable to attacks against a the few central hub genes. However, it has been recently shown that hub genes do not necessarily qualify as being fragile, and that other measures for this property might be more appropriate [64]. Analyzing the distances between vertices allows characterizing communication processes in a biological network. Therefore, we explore the distances between the vertices in the two graph prototypes. By definition, the eccentricity 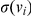 of a vertex 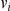 is the maximum of the shortest paths from 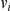 to all other vertices 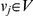. For the benign graph prototype the majority of vertices have a 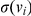 of 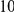, while for the cancer graph prototype the majority of vertices have a 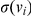 of 1. We compare the eccentricity distributions of the two networks with a Kolmogorov-Smirnov test, which results in a highly significant difference (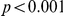). Another interesting network characteristic is the network diameter 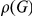, which is the maximum of all 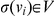. For the two graph prototypes the diameters are 17 (benign) and 12 (cancer). However, when analyzing the average path length in the largest connected components of the graphs we find it to be 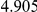 for the benign graph prototype and 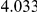 for the cancer graph prototype. Furthermore, we see only a small difference in the average clustering coefficients 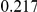 (benign) and 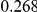 (cancer), which is the mean of the local clustering coefficient [65].

**Table 5:** Distribution of main hubs in graph protoypes.

benign															
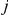	107	105	96	91	88	87	86	85	84	83	82	79	77	75	74
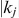	1	1	1	1	2	3	1	1	1	2	2	1	2	3	2

We list the distribution of the 15 main hub gene degrees in the two graph prototypes. Here, 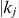 is the number of genes with 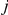 neighbors.

## Discussion

In this paper we applied a method for selecting prototypical networks for two sets of biological networks. One set of networks was based on prostate cancer data, and the other set on data from benign samples. We employed a selection of five distance measures for the task of selecting the group-specific graph prototype. The first method was a classical graph distance measure [46], while the other four were based on using an information-theoretic approach [47]. We then compared the distances from all networks in the cancer group with the distances in the benign group for all five distance measures by a Wilcoxon test (see Table 4).

When applying the graph prototype method the interpretation of the results is intricate. It is necessary to understand what kind of information is captured by a graph distance measure, in order to interpret the selection of the graph prototypes. For instance for *pGED* we conclude that the graph prototype is the graph that in average needs the least number of morphological operations to reach morphological equivalence to all other graphs. Interpreting the *KLD*-based results is more difficult since they withhold direct information on the underlying measures, but refer to the distances between the distributions of topological properties. So, the gained information tells about the distances between the distributions for the used topological network measures but does not allow for direct interpretation of the underlying topological network measures themselves. Most of the existing graph similarity measures are either computationally demanding (NP-complete in the case of unlabeled graphs) or expect the graphs to be uniquely labeled in order to ensure efficient computation. Three of the presented information-theoretic distance measures do not rely on the graphs to be labeled uniquely, but still demonstrate acceptable computational performance, as they rely solely on the distribution of the underlying features. This also effects the phase of data preparation and network inference. Here, we had to first map the microarray-specific probe-set ids to a common identifier (Entrez gene), and then infer the underlying networks for *pGED* and 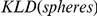 to work efficiently. In other cases, where a distance measure is applied that is independent of vertex labels, no mapping is required. This issue is of importance with respect to classical network meta-analysis methods that are based on counting common edges [10], [19] or summarizing the effect-sizes of common edges [20]. Then, the identification of common edges is a crucial requirement for the employed methods. In the present paper, we demonstrated an approach that is in principle independent of this requirement.

To investigate a potential systematic effect caused by the cancer, we performed a Wilcoxon test on the set of distances between cancer and benign networks. The results indicate that a systematic effect is likely to be present as we can see significant differences (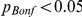) for three out of five graph distance measures. When considering the 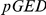 as a gold standard distance measure, we can find the following: Firstly, there is a statistically significant difference between the distances in the benign data and the cancer data (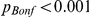), and secondly these distances are much smaller within the cancer data. Additionally, the two networks are selected as graph prototypes that are selected by most other distance measures as well. We also observe that within the benign data two clusters are formed, as can be seen in the boxplots for the measures 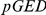, 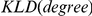, 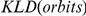, and 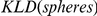. Our observation is also reflected by the hierarchical clustering (Fig. 9). The three benign networks (Chandran, Liu, Wallace) that form a cluster of their own have a fairly small sample size (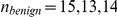). This might be the main reason for the clustering result. However, this needs to be further validated with additional data in future studies. We demonstrated in previous work that complex quantitative graph measures are capable of capturing differences in the underlying topology of biological networks for prostate cancer samples and benign samples [66]. This indicates that the cancer causes functional changes to sets of genes that are reflected in changes of structural properties. One structural change that we observed is related to the degree distribution, as the hub genes in the cancer graph prototype are remarkably smaller than those in the benign graph prototype. The topological analysis of the graph prototypes leads us to hypothesize that the prostate cancer is rewiring the communication paths in the diseased cells. By intuition it is possible to assume that the flow of information takes longer in networks with a larger diameter [67]. A topological analysis of different signaling networks by Schramm et al. led to similar results for prostate cancer and cancer in general [68]. They observed a slight decrease in the average path length for cancer networks and the tendency to form hubs was lower in cancer networks [68]. Schramm et al. also observed a decrease in local clustering coefficients for cancer networks [68], which we could not observe in our graph prototypes. However, in their networks for prostate cancer one network exhibited a small increase in the local clustering coefficient, so this calls for a further analysis. As they investigated one network from the same data we did select as the cancer graph prototype (Wang [34]), the overlap in the results is no surprise. Still, by graph prototyping we came to similar conclusions with respect to cancer networks as they did in their study. For a topological analysis, Wang et al. investigated the role of hub genes in aggressive forms of prostate cancer [61]. They observed dysregulations in genes that are related to the cell-cycle [61]. Our goal is now to identify further structural changes they might be used as markers for disease-specific events.

Taking the sample size of the single studies into account shows that four out of five times the network from the largest study (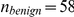) was selected as graph prototype for the benign data. In the case of the cancer data the network from the largest study (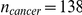) is always selected. This leads us to conclude that the network that was inferred from the largest study, represents all the other networks the best. The sensitivity regarding the sample size, which massively influences the quality of the inferred network, is also reflected by the hierarchical clustering. However, this quite intuitive hypothesis needs further verification in future work. Therefore we plan on pursuing a twofold approach: On the one hand by calculating more distance measures on the present data and, on the other hand, by testing the employed methods on new networks. This should also allow investigating how the distances are distributed in other types of cancer or even in other diseases. Interestingly, whenever a distance measure showed a significant difference between benign and cancer network distances, the same networks were selected as graph prototype: The Wang data for the cancer networks and the Yu data for the benign networks. This coherence might indicate that the more specific a used distance measure captures group (benign or cancer) information the better is works for the selection of a graph prototype.

The selected graph prototypes might be thought of as *structural* prototypes for the available set of networks. This means that, with respect to the employed distance measure, the graph prototypes represent the topological properties of the entire set of networks. Therefore, the information that is based on the topological properties of the graph prototype can be used for succeeding network analysis. However, the outcome depends directly on the quality of the set of networks. Note, that this approach always selects one network as being representative for the set, regardless of the underlying distances. The selection alone is therefore primarily no measure for the quality of a single study or the used inference method, but a result driven by the selected distance measure. The employed distance measure and the related quality of the result have to be considered in order to assess the outcome quality. An upper threshold for the average distance might be introduced to force meaningful selections, but was disregarded in this present study. A topic that has been omitted from our analysis so far is semantic similarity between networks. We expect functional similarity to be of importance when comparing biological data, and therefore plan on investigating the role of semantic relatedness in more detail. This would enable us to not only integrate topological information, but a whole set of other potential distance and similarity measures.

The presented methods provide a consistent and reproducible procedure for performing integrative network analysis. As the quality assessment for inferred networks is a challenge in systems biology, using the presented methodology helps to address this issue by quantifying inner-group and outer-group similarity in simple way. It can also be used to determine the quality of a newly inferred network, by comparing it to a set of existing networks. If the observed distances lie within a certain range of validity the network might be considered of reasonable quality or even as new prototype for this set. Whereas, if it differs strongly from all other networks it might be considered as being potentially erroneous. A possible application of this method is to assess the quality of trimming for indirect linkages or partial correlations as addressed by [42]. This could be done by performing graph prototyping before and after the trimming and comparing the two sets of networks. We are confident that in a similar manner as classical meta-analysis has now become a standard method for gene expression data, the integrative analysis of network information will become a common procedure in future systems biology applications. A broad range of methods for quantitative network analysis in this research field is currently emerging. Therefore, finding and developing methods and applications for a combined analysis is an ongoing challenge, yet open for defining standard methods and tools. Our presented method brings the advantage of being easily adoptable to other distance measures, that captures the underlying information better.

### Conclusion

It is a challenging task to infer common topological properties from a set of networks. Frequently, this is done by detecting common edges, which is, however, a burdensome procedure. Different vertex labels from different platforms make it hard to infer what edges are common. Finding common vertex labels is however often challenging. Our goal in this paper was to employ an alternative approach, that is independent from mapping vertex labels. We tackled this problem by selecting one network from a set of networks to be a representative for the complete set. This structural graph prototype was then used for succeeding topological analysis. To perform a comparative analysis thereof, we introduced four information-theoretic graph distance measures. Our initial hypothesis was that the distances between networks differ significantly between the group of prostate cancer networks and the group of benign networks. For three out of five employed graph distance measures we positively tested the initial hypothesis using a Wilcoxon test (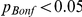). As future work, we will investigate other diseases as well and perform graph prototyping on other types of biological networks.
